# Identification of ultrasound-associated gene candidates in myeloid cells and construction of a prognostic risk model for acute myeloid leukemia

**DOI:** 10.7717/peerj.21661

**Published:** 2026-09-07

**Authors:** Lijia Zhu, Yinghua Dai

**Affiliations:** 1 Department of Ultrasound, Jiangnan Hospital Affiliated to Zhejiang Chinese Medical University, Hangzhou, China; 2 Department of Ultrasound, Hangzhou Xiaoshan Hospital of Traditional Chinese Medicine, Hangzhou, China

**Keywords:** Ultrasound, Acute myeloid leukemia, Immune cell infiltration, Single-cell RNA sequencing, Somatic mutation

## Abstract

**Background:**

Incorporating ultrasound (US) treatment sensitivity analysis may improve the treatment of acute myeloid leukemia (AML).

**Methods:**

This study integrated single-cell and bulk datasets for analysis. Differential expression analysis between US-treated and control samples was performed using limma package. The AUCell package was used to calculate US-associated scores in the single-cell dataset. Differentially expressed genes (DEGs) between the specific groups were identified, followed by intersection analysis with previously identified DEGs. Univariate regression, Least Absolute Shrinkage and Selection Operator (LASSO) analysis (using the glmnet package), and stepwise multivariate regression (using the MASS package) were used to refine the candidate genes and to construct a risk model. The model genes were validated using *in vitro* experiments. Enrichment analysis was conducted using gene set enrichment analysis (GSEA), and immune infiltration was evaluate by single-sample GSEA (ssGSEA) and ESTIMATE algorithms. The correlations between RiskScores and drug sensitivity were analyzed by oncoPredict package. Finally, tumor mutational burden (TMB) and genomic mutations were compared between the risk groups.

**Results:**

Nine prognostic signatures (*SPINK2*, *HNRNPAB*, *SH3BGRL3*, *CLEC11A*, *ITGA4*, *RPL39L*, *MX1*, *HEXIM1*, and *MAP4K4*) were identified. Particularly, low expression of *SPINK2* attenuated the activity and invasion of AML cells. High-risk group had higher immune cell infiltration. Eight drugs were predicted to be correlated with the RiskScore model. * DNMT3A* and *RUNX1* showed higher mutation frequencies in the high-risk group, whereas *KIT* and * MUC16* showed higher mutation frequencies in the low-risk group.

**Conclusion:**

The RiskScore model established in this study provides a theoretical basis for clinically screening responsive populations and optimizing treatment strategies.

## Introduction

Acute myeloid leukemia (AML) develops when myeloid blasts infiltrate the bone marrow (BM) and peripheral blood, disrupting normal blood formation and eventually leading to marrow failure. As the most common form of adult acute leukemia (Shimony, Stahl & Stone, 2023) AML continues to pose a significant clinical challenge (Marchi et al., 2025; Baden et al., 2025). Cytoreductive chemotherapy (Rausch et al., 2023), along with autologous and allogeneic hematopoietic cell transplantation, has constituted the mainstay of AML treatment. Somatic driver mutations are prevalent in AML, rendering targeted therapies, such as inhibitors of Isocitrate Dehydrogenase 1/2 (*IDH1/2*), B-cell lymphoma 2 (*BCL-2*), and FMS-related tyrosine kinase 3 (*FLT3*), critical treatment modalities (Schlenk, 2023). In Western countries, the median age at diagnosis of AML ranges from 65 to 72 years, with incidence rising markedly after age 75. Effective management remains challenging, particularly in elderly, refractory, and relapsed patients (Jaramillo & Schlenk, 2023). Therefore, accurate AML-associated biomarkers should be identified to facilitate early diagnosis, predict treatment response, and guide the development of personalized therapeutic strategies (Seyfinejad & Jouyban, 2022).

Ultrasound (US) is defined as sound waves with frequencies exceeding the human hearing threshold of 20 kHz. For therapeutic purposes, low-intensity pulsed ultrasound (LIPUS) and high-intensity focused ultrasound (HIFU) are the two main types of commonly used ultrasonic intensities (Lopez et al., 2021). Notably, LIPUS can induce sophisticated cellular and molecular immune responses by triggering inflammatory reactions, thereby enhancing cancer immunotherapy. Furthermore, LIPUS induces apoptosis in human leukemia cell lines through pathways involving *BCL-2*. Given its few side effects and easy accessibility, US holds the potential to overcome treatment failure caused by chemotherapy resistance and to synergize with immunotherapy. Therefore, investigating the effects of US on AML, and particularly its underlying mechanisms, is of both scientific and clinical importance. Such investigations may lead to improved outcomes for patients who are chemoresistant or have experienced relapse.

Traditional molecular markers often fail to account for intratumoral heterogeneity, a critical factor influencing treatment response and prognosis (Vago & Gojo, 2020). Currently, scRNA-seq can help decipher transcriptional heterogeneity within leukemic cell populations at the single-cell level (Zulibiya et al., 2023). Meanwhile, bulk RNA-seq provides population-level expression data, and the integrative analysis of these two technologies can enhance analytical robustness and clinical generalizability. Incorporating US treatment sensitivity analysis into this integrative framework facilitates the identification of key molecules closely associated with US response. Such molecules may serve as potential prognostic biomarkers or therapeutic targets, thereby aiding the combination of US with other treatment modalities (Wang, Zhao & Zhang, 2024). In this study, we further compared immune infiltration, drug sensitivity, and somatic mutations between high- and low-risk groups and validated the constructed risk model through *in vitro* experiments. Collectively, our work may help guide treatment decisions for AML patients who have become refractory to standard therapies.

## Material and methods

### Data collection

The bulk datasets were sourced from the TCGA and GEO databases. The TCGA-LAML, downloaded from the XENA database (https://doi.org/10.5281/zenodo.22104076), served as the training set. It contained transcripts per million (TPM) and clinical phenotype data for 132 AML samples.

The GEO datasets (https://www.ncbi.nlm.nih.gov/geo/) were used as validation sets: GSE10212 (two US-treated AML samples and two control samples), GSE10358 (91 AML samples), and GSE37642 (417 AML samples). The single-cell dataset GSE116256 from GEO included 16 AML patients at diagnosis and four BM aspirates from healthy donor. The analysis workflow for this study is shown in Fig. S1.

### Data preprocessing

First, bulk datasets were preprocessed by removing samples without complete survival data, ensuring survival time >0, and converting Ensembl IDs/probes to gene symbols. Next, scRNA-seq data from GSE116256 were processed subjected to quality control, in which genes detected in fewer than three cells and cells expressing fewer than 200 genes were excluded. Cells were filtered using the following criteria: nFeature_RNA >300, nCount_RNA ≤ 100,000, and mitochondrial gene percentage (percent.mt) <10%. Subsequent normalization was performed using the SCTransform function, followed by principal component analysis (PCA) with RunPCA. We corrected batch effects across different samples using the Harmony algorithm, with the following parameter settings in the RunHarmony function: group.by.vars = “orig.ident”, max.iter.harmony = 50, lambda = 0.5, and assay.use = “SCT”. The first 30 PCs were used for dimensionality reduction by Uniform Manifold Approximation and Projection (UMAP). Cell subpopulations were clustered using FindNeighbors and FindClusters functions. Finally, cell types were annotated based on marker genes from the literature (Guo et al., 2021) and the CellMarker2.0 database (http://117.50.127.228/CellMarker/).

### Identification of US-associated gene candidates from bulk RNA-seq data

To screen an initial set of ultrasound-associated genes, differential expression analysis was first performed on the bulk transcriptomic dataset GSE10212 using the limma package (Ritchie et al., 2015). The selection criteria were set as *p* < 0.05 and —logFC— > log2(1.5), yielding a total of 756 significantly dysregulated genes (Table S1). This gene set was defined as the ultrasound-associated feature gene pool for subsequent single-cell analyses.

Next, we applied the AUCell algorithm (Aibar et al., 2017) to the single-cell dataset GSE116256. The area under the curve (AUC) for the predefined gene set across the expression profile of each individual cell was calculated by AUCell, thereby assigning an “ultrasound-associated score” per cell. Based on the median AUC score (0.02629682), all cells were classified into high- and low-ultrasound-associated groups. Differentially expressed genes (DEGs) between these two groups were then identified. DEGs specifically within the myeloid cell subpopulation were further extracted and intersected with the global DEGs. The screening criteria were set as log2FC ≥ 0.25 and *p* < 0.05, ultimately yielding a set of myeloid-specific ultrasound-associated DEGs. Finally, we performed GSEA on different patient groups using the ‘gseGO’ function from the clusterProfiler package (Yu et al., 2012).

### Development of an optimal prognostic model

Univariate regression analysis (*p* < 0.01) was first carried out on these myeloid DEGs. The glmnet package in R was then used for Least Absolute Shrinkage and Selection Operator (LASSO) regression to further refine the gene set. Finally, stepwise multivariate regression analysis was conducted using the stepAIC method from the MASS package. This stepwise regression utilizes the Akaike information criterion (AIC), which balances the statistical goodness-of-fit of a model against the number of parameters used. The stepAIC function starts with the most complex model and sequentially removes one variable at a time to reduce the AIC; a smaller AIC indicates a better model, reflecting adequate fit with fewer parameters. The characteristic genes were selected based on the minimum AIC value, *i.e.,* the model with the lowest AIC was chosen as the optimal prognostic model. Using characteristic genes, an optimal risk model was formulated as follow: RiskScore = Σ*β*i ×  Expi, where Expi denotes gene expression and *β*i represents the Cox regression coefficient of the gene.

RiskScores for each sample in the dataset were calculated using the model, standardized *via* z-score, and patients were divided into low-risk and high-risk groups using a threshold of 0. Kaplan–Meier (KM) curves were generated to compare overall survival (OS) between the two groups, with statistical significance assessed by the log-rank test. The timeROC package was employed to perform receiver operating characteristic (ROC) analysis on the RiskScore for prognostic classification, and the predictive performance was evaluated for 1- to 4-year follow-up period.

### Immune infiltration analysis

Enrichment scores for 28 types of immune cells in each sample (Song et al., 2023) was calculated by single-sample GSEA (ssGSEA). Additionally, the ESTIMATE algorithm was used to analyze immune components within the tumor microenvironment (TME).

### Correlation analysis between the RiskScore and drug sensitivity

The oncoPredict R package (Maeser, Gruener & Huang, 2021) was used to predict drug IC50 values for samples in the TCGA-LAML dataset. Subsequently, Pearson correlation analysis was conducted to assess the relationship between drug sensitivity and RiskScore, with significance defined as *p* < 0.05 and —cor— > 0.3.

### Tumor mutational burden (TMB) analysis

TMB and gene mutation profiles were compared between the two risk groups. Subsequently, the top 20 genes with the highest mutation frequencies in each group were identified.

### Cell culture and transfection

DMEM medium (iCell-0001, iCell Bioscience, Shanghai, China) was employed to culture human BM stromal cells HS-5 (CL-0798, Procell, Wuhan, China). RPMI-1640 medium (iCell-0002, iCell Bioscience) was utilized to culture human AML cells HL-60 (C5236, BDBio, Hangzhou, China) and NB4 (C5114, BDBio). All the three media were added with 1% penicillin-streptomycin (P/S, iCell-15140-122, iCell Bioscience) and 10% fetal bovine serum (FBS) (iCell-0500, iCell Bioscience). Cell culture was performed at 37 °C with 5% CO_2_ (3311, ThermoFisher, Waltham, MA) in an incubator. All cells passed STR identification and were confirmed without contamination of mycoplasma.

*SPINK2* gene was used to transfect corresponding AML cells. To avoid off-target effects, two sets of specific primers were designed: si-*SPINK2* #1 (5′-TCCCTCAATTTGGTCTGTTTTCA-3′) and si-*SPINK2* #2 (5′-CTCAATTTGGTCTGTTTTCAAAA-3′). A negative control (si-NC) was also prepared (GenePharma, Shanghai, China). Transfection was performed using Lipofectamine 2000 Transfection Kit (11668027, Invitrogen, Carlsbad, CA).

### Quantitative reverse transcription PCR (qRT-PCR)

The qRT-PCR was employed to validate the transfection efficiency of the *SPINK2* gene and analyze the differential expression of prognostic genes in HS-5, HL-60, and NB4 cell lines. Total RNA was isolated from each cell sample using TRIzol reagent (15596018CN, Invitrogen), and RNA purity was assessed based on the OD260/OD280 ratio using a NanoDrop spectrophotometer (ThermoFisher Scientific, Waltham, MA). cDNA was synthesized using SuperScript™ IV Reverse Transcriptase (18090200, Invitrogen). The ABI PRISM 7000 Sequence Detection System (Applied Biosystems, Billings, MT) with 2 × SYBR Green PCR Mastermix (SR1110, Solarbio, Beijing, China) were employed to perform qRT-PCR. The cycling as conducted with initial denaturation at 95 °C for 15 min, followed by 35 cycles of 95 °C for 15 s, at 60 °C for 30 s, and 72 °C for 30 s, with a final extension at 72 °C for 10 min. The 2^−ΔΔCt^ method was utilized to calculate the relative gene expressions, which were normalized to that of *GAPDH*. *GAPDH* was selected as the internal reference gene due to its relatively stable expression in AML-related cells and tissues (Zhang et al., 2022; Brendel et al., 2015). Primer sequences for all genes are detailed in Table 1.

### CCK-8 and transwell invasion assays

AML cells in the logarithmic growth phase were planted at 5 ×10^3^ cells/well into 96-well plates. At 0, 24, 48, and 72 h after seeding, each well was supplemented with 10 µL of CCK-8 reagent (CA1210, Solarbio). Following 2-h incubation in the dark, a microplate reader (ThermoFisher Scientific, Waltham, MA) was employed to read absorbance values (OD) at 450 nm. Three biological replicates were set for each group to assess cell proliferation.

For the Transwell invasion assay, Transwell insert (8 µm, FTW010, Beyotime, Shanghai, China) was pre-coated with Matrigel (M8370, Solarbio) and loaded with AML cells suspended in non-serum medium. Complete medium containing 10% FBS was filled into the lower chamber as a chemoattractant. After incubation for 48 h, invaded cells were fixed by 4% paraformaldehyde (P1110, Solarbio) for 15 min, colored by 0.1% crystal violet (G1063, Solarbio) for 15 min, and visualized under an inverted microscope in at least three randomly selected fields (Olympus, Tokyo, Japan) (Zhang et al., 2024).

### Statistical analysis

R software (version 3.6.0) and GraphPad Prism (version 8.0.2) were employed in all data analyses. Continuous variables between groups were compared by parametric tests using two-tailed Student’s *t*-test (for variables with normal distribution), whereas Mann–Whitney U test (Wilcoxon rank-sum test) was used in non-parametric tests. For comparisons with three or more groups, analysis of variance (ANOVA) and Bonferroni’s multiple comparisons test were utilized.

**Table 1:** The primers utilized in this study.

Gene	Accession No.	Primers (5′–3′)	
		Forward	Reverse
*SPINK2*	NM_021114	GATTACCAGGATGTCCCAGACAC	TGCTCCATCAGCAGGGTCCATT
*HNRNPAB*	NM_031266	TCCCAACACTGGACGGTCAAGA	GGGTCCTTCTTCATAGCCATGG
*SH3BGRL3*	NM_031286	CAGTCTCACACTGAGCTCCAGA	CCTTCCCCCTCGGTTACCAGG
*CLEC11A*	NM_002975	ACACCCGCGATGCCGTGCAAG	CGAGAGCAGGAAGCACTTGTGG
*ITGA4*	NM_000885	GCATACAGGTGTCCAGCAGAGA	AGGACCAAGGTGGTAAGCAGCT
*RPL39L*	NM_052969	AGGCGTCTCGGAGTCTCAGA	TCCCTCAGGTCTGCGTGTCA
*MX1*	NM_002462	GGCTGTTTACCAGACTCCGACA	CACAAAGCCTGGCAGCTCTCTA
*HEXIM1*	NM_006460	GAGGACAGTAGGTGGCAATCGA	AGGCAGCTAGATTCTGGACAGG
*MAP4K4*	NM_145686	CCAATGGCAACTCCGAGTCTGT	GGGTCACTGAAGGAATGGGATC
*GAPDH*	NM_002046	GTCTCCTCTGACTTCAACAGCG	ACCACCCTGTTGCTGTAGCCAA

## Results

### Identification of US-associated genes

**Figure 1:**
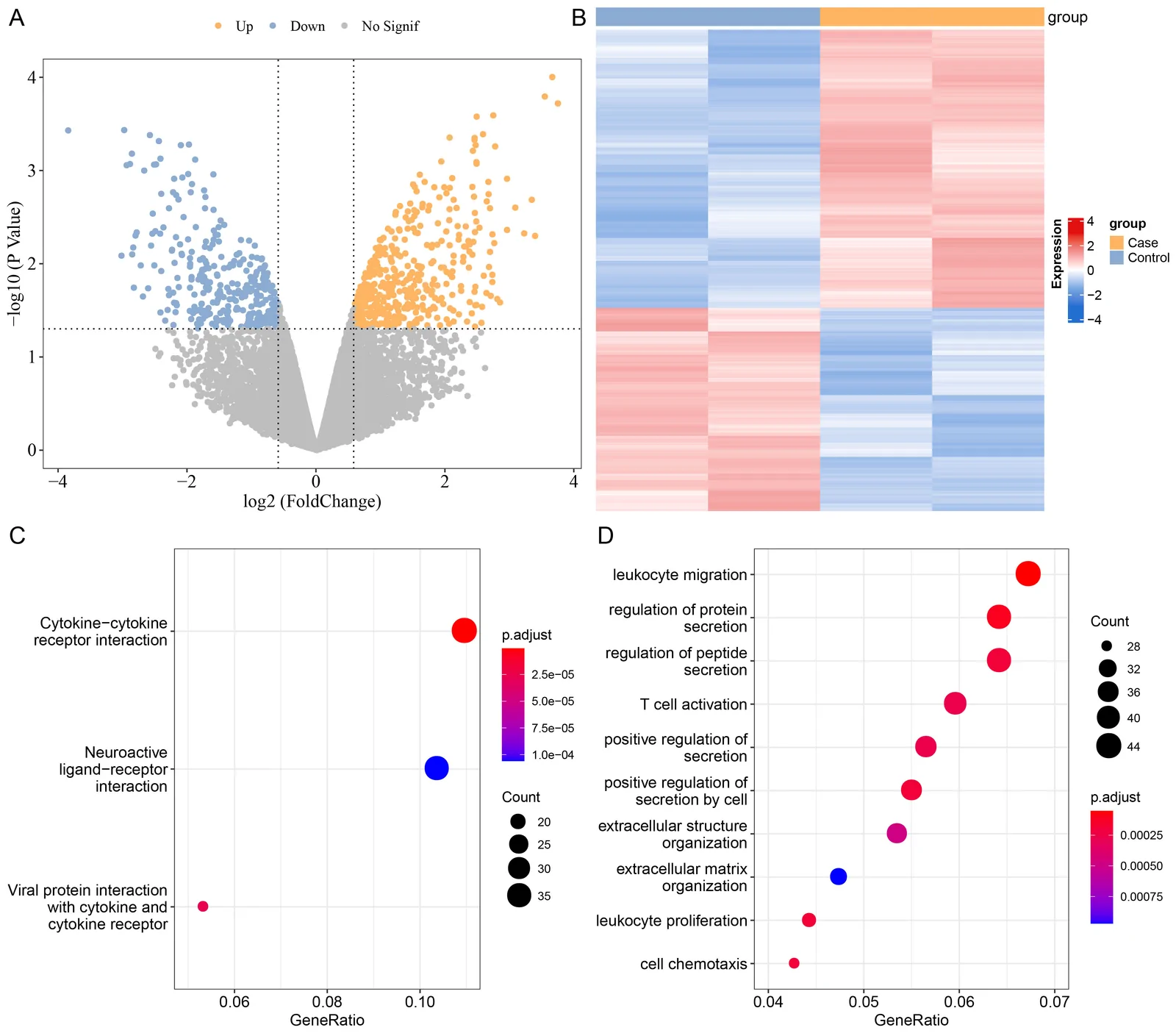
Identification and analysis of DEGs between US-treated and control groups. (A) Volcano plot of DEGs between US-treated and control groups. (B) Heatmap of DEGs between US-treated and control groups. (C) KEGG pathway enrichment results of DEGs. (D) GO biological process enrichment results of DEGs.

Differential expression analysis between US-treated and control samples in the GSE10212 dataset yielded 756 significantly DEGs, including 319 downregulated and 437 upregulated genes (Figs. 1A–1B). Functional Kyoto Encyclopedia of Genes and Genomes (KEGG) pathway and GO analyses revealed that these DEGs were significantly enriched in pathways, including leukocyte migration and cytokine-cytokine receptor interaction (Figs. 1C–1D).

### Single-cell atlas of AML

**Figure 2:**
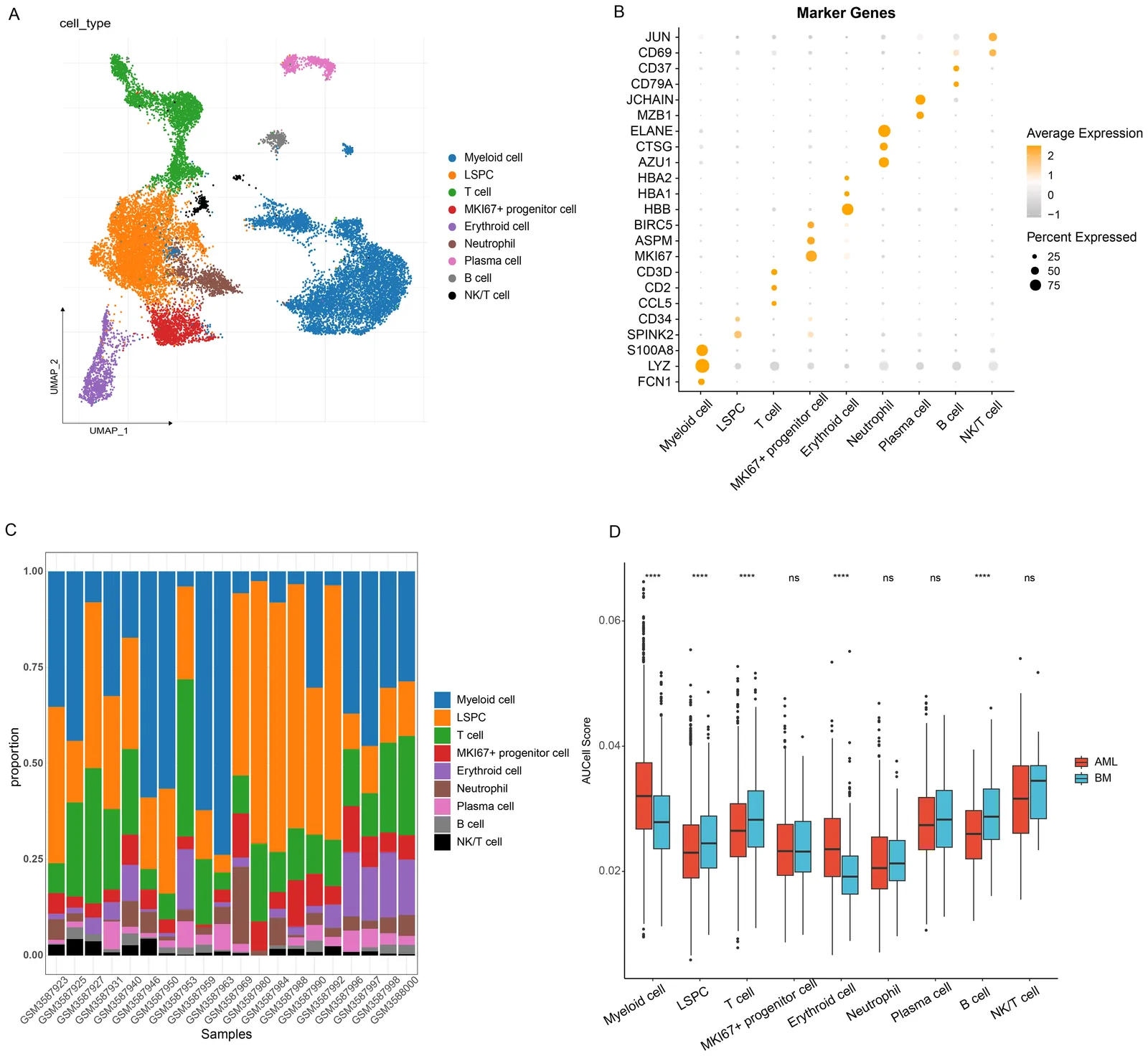
Single-cell atlas of GSE116256 dataset. (A) UMAP visualization of annotated cell clusters. (B) Bubble plot showing the expression levels of canonical marker genes across nine cell subsets. (C) Proportions of cell subsets in each sample from GSE116256. (D) Boxplots comparing AUCell enrichment scores of the ultrasound–associated gene set across different cell types between AML and BM groups. The boxplot center line indicates the median, box bounds represent the interquartile range (IQR), and whiskers extend to the largest/smallest values within 1.5 × IQR. Statistical comparisons between AML and BM groups for each cell type were performed using the two–sided Wilcoxon rank–sum test. Significance levels are indicated as: ns means non-significant, **** means *p* < 0.0001.

Following preprocessing of the scRNA-seq data from GSE116256, a total of 20,323 high-quality cells were retained and clustered into 14 distinct cell groups (Fig. S2). Based on the expression patterns of canonical marker genes, these clusters were further annotated into nine major cell types, including myeloid cells, leukemia stem/progenitor cells (LSPCs), T cells, MKI67^+^ progenitor cells, erythroid cells, neutrophils, plasma cells, B cells, NK and T cells (Figs. 2A–2B). Among these, myeloid cells and LSPCs exhibited the highest proportions in the cellular composition (Fig. 2C). Subsequently, AUCell enrichment scores for US-associated genes were calculated across all cell types. Notably, myeloid cell subsets in AML samples showed significantly higher AUCell scores compared to BM samples (Fig. 2D, *p* < 0.05).

### Identification of US-associated key genes in myeloid cells

**Figure 3:**
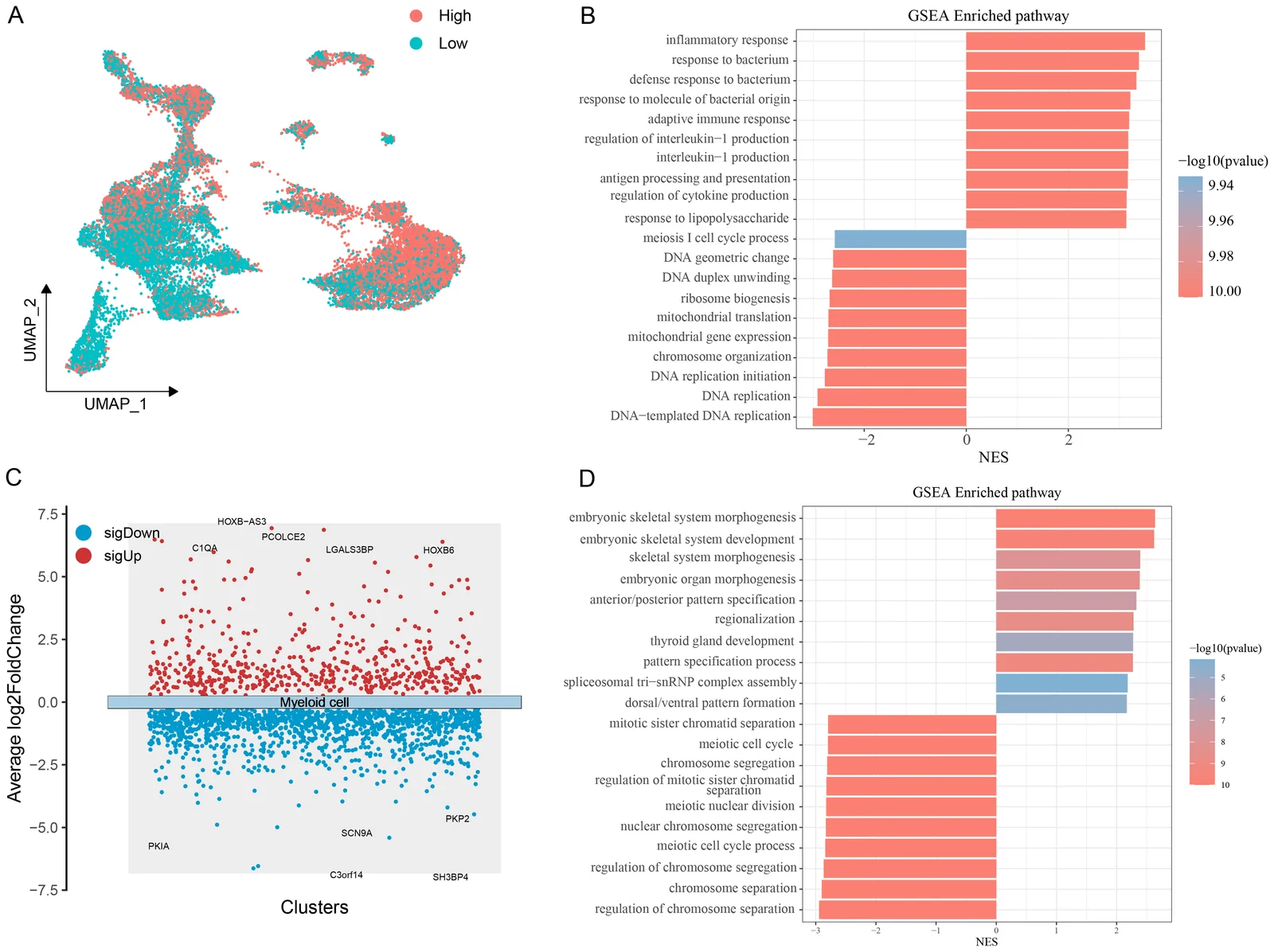
Identification of US-sensitive genes in myeloid cells. (A) UMAP projection showing enrichment scores of US-sensitive genes in individual cells. Red indicates the high-US-sensitive group, and blue indicates the low-US-sensitive group. (B) Differences in biological processes between high- and low-US-sensitive groups. Normalized enrichment score (NES) > 0 indicates significant enrichment of gene sets in the high-US-sensitive group, whereas NES < 0 indicates significant enrichment in the low-US-sensitive group. (C) Screening of DEGs in myeloid cells. (D) Differences in biological processes between AML and control (BM) groups in myeloid cells. NES > 0 indicates significant enrichment of gene sets in the AML group, and NES < 0 indicates significant enrichment of gene sets in the control (BM) group.

US-associated scores were calculated for all cells, and cells were stratified into high- and low-US-associated groups by median scores (Fig. 3A). Differential expression analysis between the two groups identified 2,978 downregulated and 1,358 upregulated genes. High-US-associated group exhibited activation of biological processes such as inflammatory response and response to bacterium, while low-US-associated group showed enrichment in DNA replication, DNA duplex unwinding and (Fig. 3B). Subsequent analysis of myeloid-specific DEGs identified 1,375 downregulated and 551 upregulated genes (Fig. 3C). GSEA in myeloid cells from AML samples revealed activation of pathways such as embryonic skeletal system morphogenesis (Fig. 3D). Intersection of myeloid DEGs with global US-associated DEGs yielded 1,232 genes, which were defined as US-associated DEGs in myeloid cells.

### Development and validation of a clinical prognostic model

**Figure 4:**
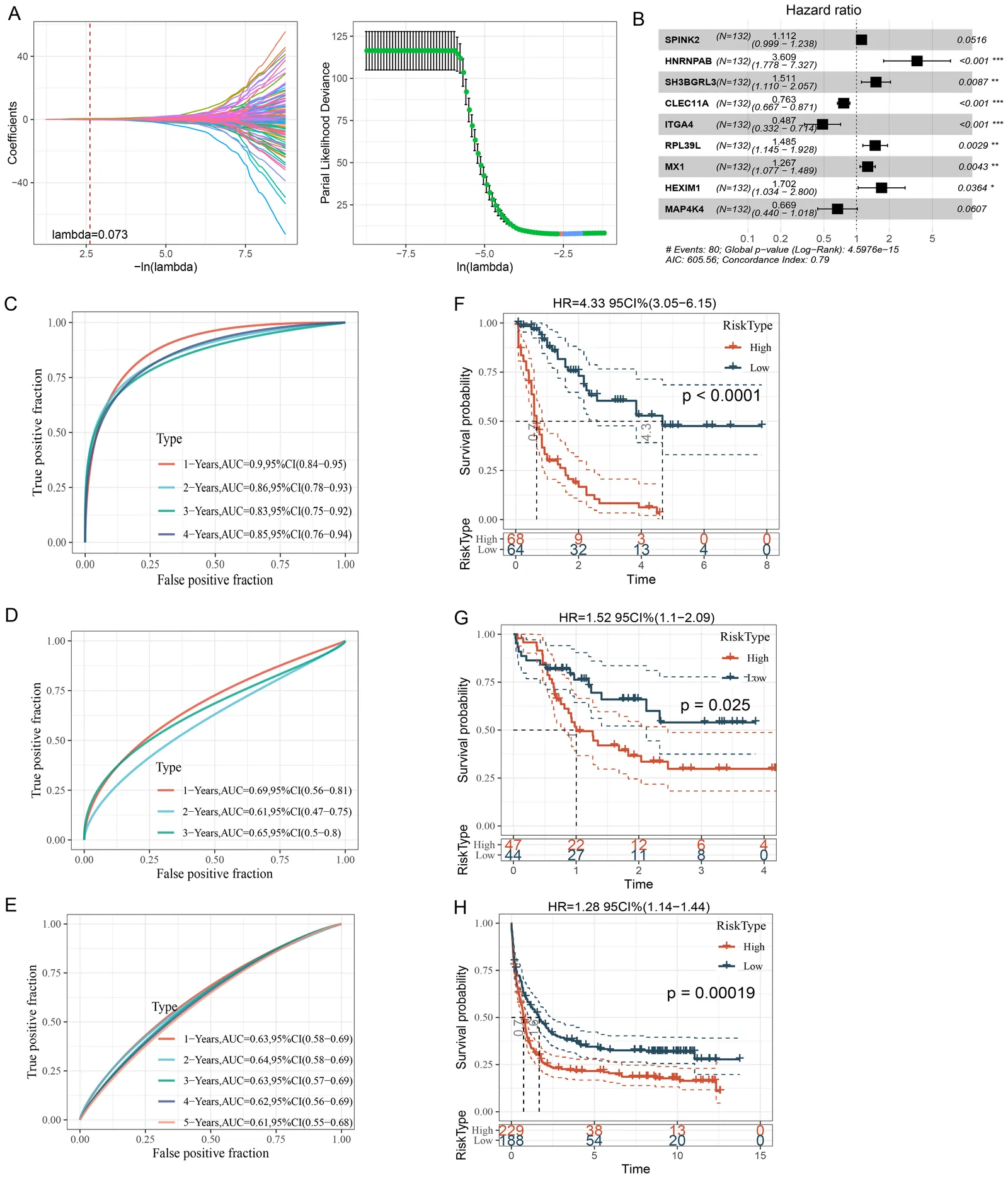
Construction and validation of the clinical prognostic model. (A) Coefficient trajectories of prognostic variables across different lambda values and confidence intervals at the optimal lambda. (B) Forest plot depicting hazard ratios of signatures in the prognostic model. And * means *p* < 0.05, ** means *p* < 0.01, *** means *p* < 0.001. (C–E) Performance assessment of the prognostic model in (C) TCGA-LAML, (D) GSE10358, and (E) GSE37642 cohorts. (F–H) KM survival curves comparing high- and low-risk groups in (F) TCGA-LAML, (G) GSE10358, and (H) GSE37642 datasets.

Univariate Cox regression analysis was performed for the 1,232 US-associated DEGs in myeloid cells, identifying 109 genes with prognostic significance (*p* < 0.01). LASSO regression was applied to further shrink these prognosis-associated genes. A set of 19 genes with *λ* = 0.073 was selected for subsequent analysis (Fig. 4A). Stepwise multivariate regression analysis based on the AIC was then used to derive the final model. Ultimately, nine genes were identified for developing a prognostic model (Fig. 4B) as follow: RiskScore = 0.106*SPINK2 +1.284*HNRNPAB +0.413*SH3BGRL3 −0.271*CLEC11A −0.719*ITGA4 +0.396*RPL39L +0.236*MX1 +0.532*HEXIM1 −0.402*MAP4K4.

Using this formula, RiskScore was calculated for all samples and standardized by z-score, resulting in 68 samples classified into the high-risk group and 64 into the low-risk group. ROC analysis for 1- to 4-year prognostic prediction showed a high AUC values for the model (Fig. 4C). Similar results were observed in the validation datasets GSE10358 and GSE37642 (Figs. 4D–4E). According to survival analysis, low-risk patients exhibited notably better OS than the high-risk patients (*p* < 0.05, Figs. 4F–4H).

### *In vitro* validation of the prognostic model

**Figure 5:**
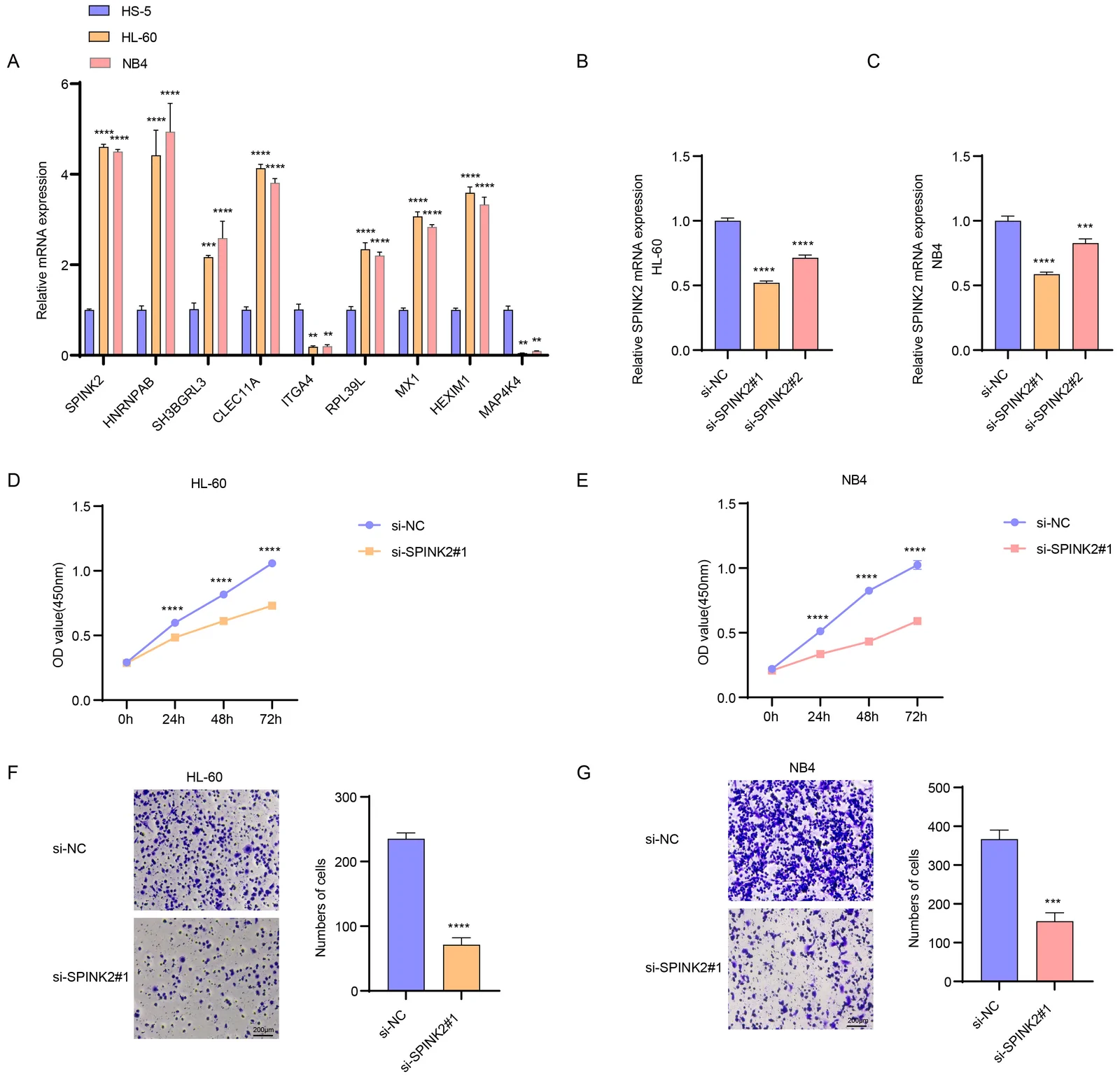
*In vitro* experimental results. (A) Expression levels of nine prognostic signatures (SPINK2, HNRNPAB, SH3BGRL3, CLEC11A, ITGA4, RPL39L, MX1, HEXIM1, MAP4K4) in HS-5, HL-60, and NB4 cell lines. (B) Expression levels of SPINK2 in HL-60 cells transfected with si-NC, si-SPINK2#1, and si-SPINK2#2. (C) Expression levels of SPINK2 in NB4 cells transfected with si-NC, si-SPINK2#1, and si-SPINK2#2. (D) Cell viability comparison between si-NC and si-SPINK2#1 groups in HL-60 cells. (E) Cell viability comparison between si-NC and si-SPINK2#1 groups in NB4 cells. (F) Cell invasion ability comparison between si-NC and si-SPINK2#1 groups in HL-60 cells. (G) Cell invasion ability comparison between si-NC and si-SPINK2#1 groups in NB4 cells. ** means *p* < 0.01, *** means *p* < 0.001, **** means *p* < 0.0001.

*In vitro* results showed that, except for *ITGA4* and *MAP4K4*, which were significantly downregulated in two AML cell lines (HL-60 and NB4), the remaining seven genes (*SPINK2*, *HNRNPAB*, *SH3BGRL3*, *CLEC11A*, *RPL39L*, *MX1*, and *HEXIM1*) were all significantly upregulated in AML cell lines (*p* < 0.05, Fig. 5A). *SPINK2* was randomly selected for further transfection, and successful transfection was confirmed. The si-*SPINK2* #1 with higher transfection efficiency was chosen for subsequent experiments (*p* < 0.05, Figs. 5B–5C). CCK-8 assay demonstrated that the cell viability of the si-NC group was significantly higher than those of the si-*SPINK2* #1 group (*p* < 0.05, Figs. 5D–5E). Transwell assay showed that the cell invasion ability of the si-NC group was notably higher than that of the si-*SPINK2* #1 group (*p* < 0.05, Figs. 5F–5G). These findings indicated that low expression of the *SPINK2* gene can weaken the activity and invasion ability of AML cells.

### Analysis of the risk model with immune microenvironment and chemosensitivity

**Figure 6:**
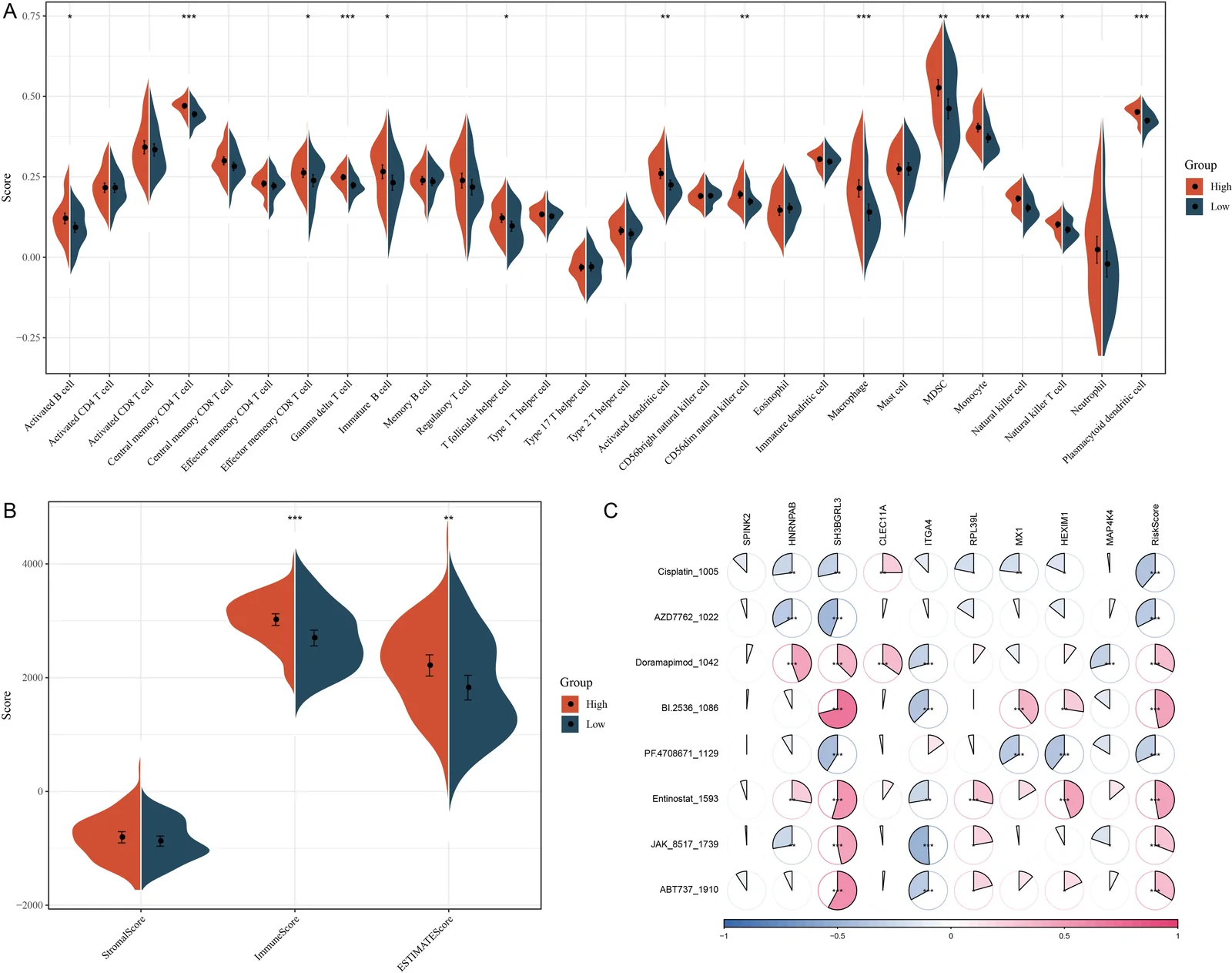
Analysis of immune infiltration and drug sensitivity. (A) The ssGSEA-based assessment of differences in 28 immune cell scores between high- and low-risk groups. (B) ESTIMATE analysis of immune cell infiltration in high- and low-risk groups of the TCGA-LAML cohort. (C) Correlation analysis between RiskScore, prognostic signatures expression in the model, and drug IC50 values in the TCGA-LAML dataset. Red indicates positive correlation, while blue indicates negative correlation. * means *p* < 0.05, ** means *p* < 0.01, *** means *p* < 0.001, **** means *p* < 0.0001.

The ssGSEA results showed that the abundance of most immune cells, such as activated B cells, immature B cells, macrophages, and monocytes, was significantly higher in the high-risk group (Fig. 6A, *p* < 0.05). ESTIMATE algorithm analysis revealed that both ImmuneScore and ESTIMATEScore were significantly higher in the high-risk group (Fig. 6B, *p* < 0.05). To examine the relationship between the model and drug sensitivity, eight drugs were identified as having significant correlations with the RiskScore. Specifically, Doramapimod_1042, BI.2536_1086, Entinostat_1593, JAK_8517_1739, and ABT737_1910 were more suitable for the low-risk group, while Cisplatin_1005, AZD7762_1022, and PF.4708671_1129 were more suitable for the high-risk group (Fig. 6C, *p* < 0.05).

### Mutational differences across RiskScore groups

**Figure 7:**
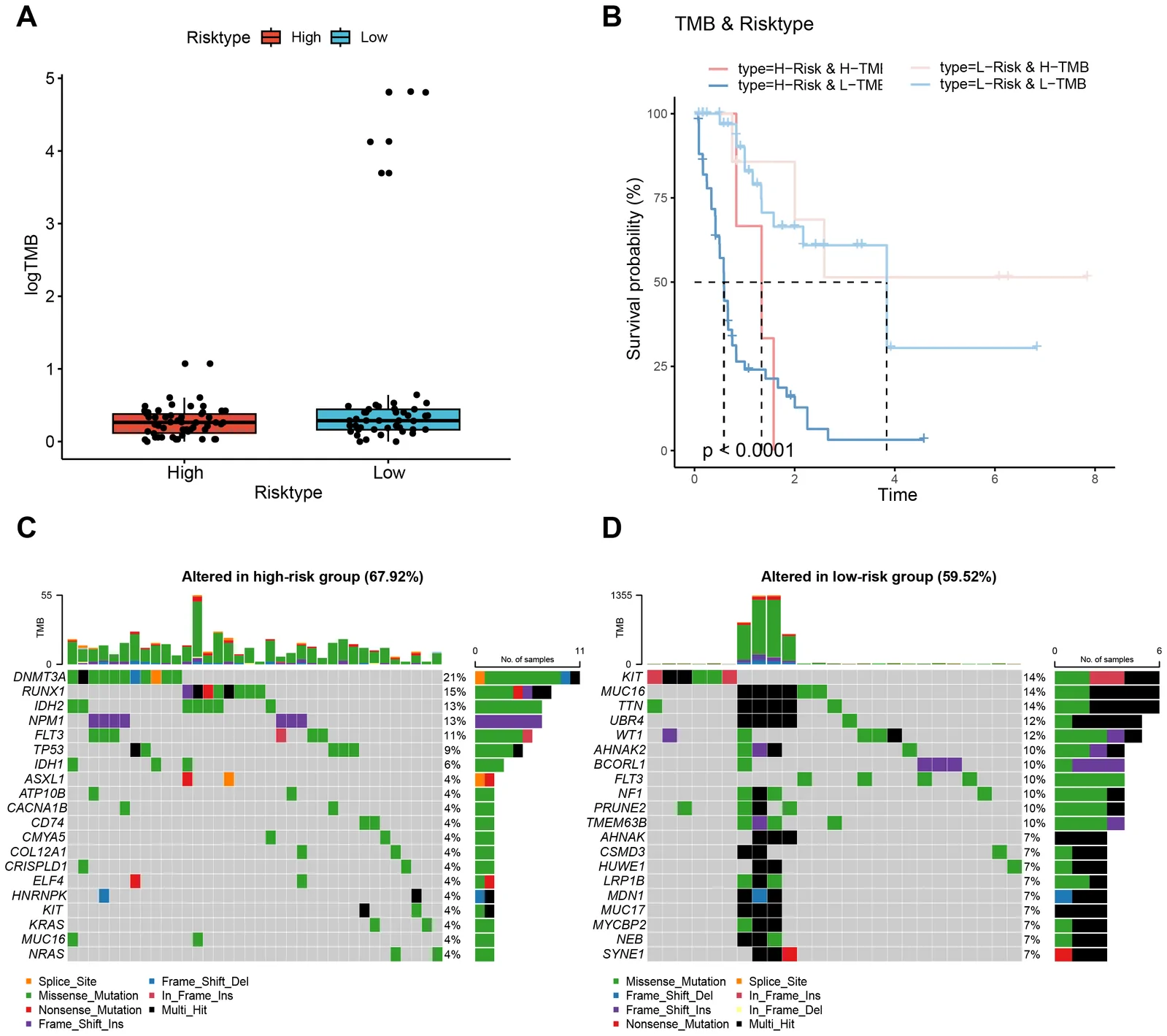
TMB and frequently mutated genes between risk groups. (A) Differences in TMB between high- and low-risk groups. (B) KM curves for combined TMB and RiskScore stratification in the TCGA-LAML cohort. (C–D) Top 20 frequently mutated genes in (C) high-risk and (D) low-risk groups of the TCGA-LAML cohort.

We first compared the tumor mutational burden (TMB) between the high-risk group and the low-risk group and found no significant difference in TMB between the two groups (*p* > 0.05, Fig. 7A). Synergy analysis between TMB and US revealed that the low-risk group had consistently better prognosis than those in the high-risk group within both high-TMB and low-TMB subgroups (Fig. 7B), indicating that TMB status did not interfere with the predictive efficacy of the RiskScore. Comparison of genomic mutation profiles showed that the mutation frequencies of *DNMT3A* and *RUNX1* were higher in the high-risk group (Fig. 7C), while those of *KIT* and *MUC16* mutations were higher in the low-risk group (Fig. 7D).

## Discussion

AML is a heterogeneous hematopoietic tumor primarily treated with intensive chemotherapy (cytarabine + anthracycline, “7+3”) and hypomethylating agents. However, these regimens often results in poor outcomes and high relapse rates (Liu, 2021). US-based combination therapies can enhance tumor responsiveness to chemotherapy and immunotherapy. This study integrated single-cell RNA-seq and bulk transcriptomic data to construct a prognostic model incorporating nine prognostic biomarkers: *SPINK2*, *HNRNPAB*, *SH3BGRL3*, *CLEC11A*, *ITGA4*, *RPL39L*, *MX1*, *HEXIM1*, and *MAP4K4*. Stratification into high- and low-risk groups based on this model facilitates personalized medication selection and the investigation of gene mutation patterns. Collectively, our results provide a theoretical foundation for identifying responsive populations and optimizing treatment strategies, while also informing the development of personalized AML therapy.

Here, we established a model of AML myeloid cells related to US therapy and identified nine key biomarkers, including *SPINK2*, *HNRNPAB*, *SH3BGRL3*, *CLEC11A*, *ITGA4*, *RPL39L*, *MX1*, *HEXIM1*, and *MAP4K4*. *SPINK2*, a serine peptidase inhibitor Kazal type 2, is a classic trypsin inhibitor that has been initially discovered to be associated with fertility. Its upregulated expression in AML is linked to increased treatment resistance and relapse rate in AML patients (Chen, 2024). *H* eterogeneous nuclear ribonucleoprotein A/B (*HNRNPAB*) can regulate epithelial-mesenchymal transition (EMT) of tumor cells by influencing transcription of long non-coding RNAs (lncRNAs) and is dysregulated in multiple tumors (Wang et al., 2023). SH3 domain-binding glutamate-rich protein 3 (*SH3BGRL3*) generates circular RNAs (circRNAs) through exon circularization, and its high expression is associated with a worse OS in AML patients (Yang et al., 2023). In kidney renal clear cell carcinoma (KIRC), it encodes TNF-α inhibitory protein (TIP-B1) to regulate AKT signaling *via* the TIP-B1/ EGFR axis. C-type lectin domain-containing protein 11A (*CLEC11A*) shows a high expression in BM and regulates hematopoietic differentiation and balance (Swanson et al., 2024). The expression of *CLEC11A* exhibits discrepancies between *in vitro* experiments and our risk model. Previous research indicates that *CLEC11A* is highly expressed in AML (Mumme et al., 2023), consistent with our *in vitro* experimental results. Integrin subunit α4 (*ITGA4*) mediates the adhesion of AML cells to BM stroma and promotes migration (Chen et al., 2025). Methyltransferase-like 3 (*METTL3*) prolongs the half-life of its mRNA through m^6^A methylation, while friend leukemia virus integration 1 (*FEV*) maintains the homing and expansion of primary and relapsed AML by activating its transcription. Ribosomal protein L39 like (*RPL39L*) shares 92% protein sequence similarity with *RPL39* (Banerjee et al., 2024), a candidate gene for chronic myeloid leukemia (CML) (Alsamman et al., 2023). Myxovirus resistance 1 (*MX1*) encodes an interferon-induced guanosine triphosphatase (GTPase) (Caielli et al., 2024). Research suggests that *MX1* promotes angiogenesis and chemotherapy resistance in colorectal cancer, showing the potential to serve as a therapeutic target (Hou et al., 2025). Hexamethylene bisacetamide-inducible protein 1 (*HEXIM1*) is a transcription regulator essential for erythropoiesis (Lv et al., 2023). It activates the bromodomain and extra-terminal domain (BET) pathway through autophagic degradation in specific AML subsets, and its induced expression is involved in the anti-AML activity of BET protein bromodomain antagonists. Mitogen-activated protein kinase 4 (*MAP4K4*) is crucial for myeloid differentiation. It is expressed at higher levels in normal hematopoietic and lymphoid tumors than in AML tumors (Bai et al., 2019). Furthermore, prior studies have identified *CLEC11A* and *ITGA4* as pivotal ligand-receptor (LR) nodes in AML, which collectively constitute the core components of the LR.score and exert coordinated effects in disease progression (Fu et al., 2024). Given that they were initially identified as transcriptionally responsive to ultrasound exposure, they may serve as potential mediators of US-related biological effects in AML. Further functional studies are warranted to elucidate whether and how these genes contribute to the cellular response to US therapy.

In this study, enrichment analysis revealed that US-associated genes were significantly enriched in pathways such as leukocyte migration and cytokine-cytokine receptor interaction, consistent with the activation of the inflammatory response pathway observed in the high-US-score group. The above findings suggest that US may influence the occurrence and development of AML by regulating the inflammatory microenvironment. Inflammation, a classic hallmark of cancer, is centrally regulated by cytokines (*e.g.*, interleukin-18, IL-18), which mediate intercellular communication and play critical roles in host defense against infections and the coordination of innate-adaptive immune responses (Ihim et al., 2022). Previous studies have demonstrated the potential immune-activating potential of US therapy (Liu et al., 2024). By inducing immunogenic cell death (ICD) in AML cells and promoting the release of inflammatory cytokines, US enhances antigen presentation processes, thereby maximizing anti-tumor immune effects (Cai et al., 2025). In the low-US-associated group, enrichment of DNA replication-related pathways indicates a highly proliferative state of these cells. Conventional chemotherapy with cytarabine combined with anthracyclines (*e.g.*, daunorubicin) can effectively target and eliminate leukemic cells with high proliferative indices by inhibiting DNA polymerase and topoisomerase II (Kantarjian et al., 2025). Furthermore, immune infiltration analysis revealed significantly higher expressions of most immune cells in the high-risk group, further suggesting the need for differential treatment strategies. Specifically, the high-risk group may benefit from US combined with immunotherapy targeting the activated inflammatory microenvironment and infiltrating immune cells, while the low-risk group may be more suitable for taking traditional cytotoxic therapy centered on chemotherapy. It is important to recognize that the enrichment findings and the observed differences in immune infiltration were exploratory and did not directly establish causal relationships. The drug sensitivity results, which identified eight compounds with variable mechanisms (*e.g.*, kinase inhibition), lacked a clear convergent biological rationale. As such, the current results should be viewed as hypothesis-generating, and further experimental validation is needed to determine whether they reflect a coherent therapeutic vulnerability linked to the ultrasound-associated gene model.

Somatic mutations are known drivers of AML progression (Egan & Schimmer, 2023). As a genetically heterogeneous disease, identifiable somatic mutations are detected in 97.3% of AML cases, with the most commonly altered genes including *RUNX1*, *IDH1*, *NPM1*, *IDH2*, *FLT3*, *DNMT3A*, *NRAS*, *TET2*, and *TP53* (Venugopal & Sekeres, 2024). This study showed that *DNMT3A* and *RUNX1* exhibited higher mutation rates in high-risk group patients, while typical mutant genes such as *IDH2*, *NPM1*, and *FLT3* also showed significantly increased mutation frequencies. In contrast, *KIT* and *MUC16* were more frequently mutated in low-risk group patients. *DNMT3A* encodes a DNA methyltransferase, and its abnormal-mediated DNA methylation, particularly at cytosine-phosphate-Guanine (CpG) islands in gene promoters, is closely linked to cancer pathogenesis. Large-scale parallel sequencing has revealed recurrent *DNMT3A* mutations in patients with de novo AML, which are independently associated with poor outcomes (Kayser & Levis, 2023). *RUNX1* regulates normal and malignant hematopoiesis and is a commonly mutated gene in AML (Klco & Mullighan, 2021). Functionally, *RUNX1* interacts with *DNMT3A*; studies have proposed a model in which *RUNX1* recruits *DNMT3A* to target genes for epigenetic regulation (Liu et al., 2021). BET protein inhibitors or degraders have been shown to significantly improve survival in AML mouse models transplanted with mutant *RUNX1* (mt*RUNX1*)-expressing cells by inhibiting *RUNX1* and its downstream targets to induce apoptosis. *KIT* has recently been identified as a mutation target in AML (Katagiri et al., 2022), whereas *MUC16* mutation is relatively rare in AML. In summary, the striking differences in somatic mutation profiles between the risk groups provide critical evidence for molecular stratification, prognostic assessment, and personalized treatment of AML.

This study has certain limitations. First, the initial screening of candidate genes associated with US was based solely on two treated and two control samples, resulting in severely limited statistical power. Future validation using larger cohorts of treated samples (*e.g.*, prospective clinical trials or multi-time-point sampling in animal models) is needed to confirm whether these genes genuinely respond to US treatment. Second, the ultrasound sensitivity of the nine genes in the risk model has not been validated through direct *in vitro* ultrasound stimulation experiments. Therefore, future studies should conduct dynamic expression analysis following ultrasound irradiation in AML cell lines to distinguish between genes associated with prognosis and those responsive to ultrasound therapy. Third, *in vitro* functional validation was limited to a single gene *SPINK2* and only validated its effects on cell proliferation and invasion, without involving ultrasound treatment. Future studies should conduct functional experiments combining knockdown or overexpression of the other eight genes with ultrasound exposure, and evaluate the therapeutic efficacy of ultrasound combined with targeting these genes in *in vivo* xenograft models. Fourth, the drug sensitivity analysis was based on expression-based inference and exhibited high heterogeneity across drug classes, lacking a unified pathway logic. Future studies could employ CRISPR screening or high-throughput drug combination screening, integrated with phosphoproteomics, to identify compounds that synergize with US-associated genetic features and their shared signaling pathways. Finally, none of the inferences regarding US combined with immunotherapy have been validated *in vivo*. An AML mouse model should be established to directly compare the effects of US alone, immunotherapy alone, and their combination on tumor burden, the immune microenvironment, and patients’ survival, thereby providing empirical evidence for clinical translation. Through these multifaceted follow-up studies, the limitations of the current work can be systematically addressed, and a more solid foundation can be laid for the clinical application of this risk model.

## Conclusion

In this study, we developed a nine-gene risk model for AML. This model revealed distinct patterns of immune cell infiltration, drug sensitivity, and somatic mutation profiles between the high- and low-risk groups. These findings suggest that the risk model may be useful for stratifying AML patients and could provide a molecular basis for personalized treatment. With further validation, the RiskScore may assist clinicians in prognosis prediction and medication selection. In addition, the model revealed a potential direction for exploring novel therapeutic strategies, such as combining US with immunotherapy, although this remains to be tested in future studies.

##  Supplemental Information

10.7717/peerj.21661/supp-1 Supplemental Information 1 Flowchart of the analysis process for this study

10.7717/peerj.21661/supp-2 Supplemental Information 2 Characteristics of s amples GSE116256
(A) The nFeature_RNA i n GSE116256. (B) The nCount_RNA i n GSE116256. (C) The percent.mt i n GSE116256.Seurat_clusters in GSE116256.

10.7717/peerj.21661/supp-3 Supplemental Information 3 Identify genes associated with ultrasound therapy based on the GSE10212 dataset

10.7717/peerj.21661/supp-4 Supplemental Information 4 MIQE checklist

## Abbreviations

AML: acute myeloid leukemia
FLT3: FMS-related tyrosine kinase 3
IDH1/2: Isocitrate Dehydrogenase 1/2
BCL-2: B-cell lymphoma 2
US: ultrasound
HIFU: high-intensity focused ultrasound
LIPUS: low-intensity pulsed ultrasound
TCGA: The Cancer Genome Atlas
GEO: Gene Expression Omnibus
TPM: transcripts per million
scRNA-seq: single-cell RNA sequencing
BM: bone marrow
PCA: principal component analysis
UMAP: Uniform Manifold Approximation and Projection
DEG: differentially expressed gene
FC: Fold Change
LASSO: Least Absolute Shrinkage and Selection Operator
AIC: Akaike information criterion
KM: Kaplan–Meier
OS: overall survival
ROC: receiver operating characteristic
GSEA: gene set enrichment analysis
ssGSEA: single-sample gene set enrichment analysis
TME: tumor microenvironment
TMB: tumor mutational burden
DMEM: Dulbecco’s Modified Eagle’s Medium
RPMI-1640: Roswell Park Memorial Institute 1640 Medium
FBS: fetal bovine serum
P/S: penicillin-streptomycin
STR: Short Tandem Repeat
qRT-PCR: quantitative reverse transcription PCR
CCK-8: cell counting kit-8
ANOVA: analysis of variance
KEGG: Kyoto Encyclopedia of Genes and Genomes
GO: Gene Ontology
LSPC: leukemia stem/progenitor cell
NK: Natural Killer
AUC: area under the curve
SPINK2: serine peptidase inhibitor Kazal type 2
HNRNPAB: heterogeneous nuclear ribonucleoprotein A/B
EMT: epithelial-mesenchymal transition
lncRNA: long non-coding RNA
SH3BGRL3: SH3 domain-binding glutamate-rich protein 3
circRNA: circular RNA
TIP-B1: TNF-α inhibitory protein
EGFR: epidermal growth factor receptor
KIRC: kidney renal clear cell carcinoma
CLEC11A: c-type lectin domain-containing protein 11A
ITGA4: integrin subunit α4
METTL3: methyltransferase-like 3
FEV: friend leukemia virus integration 1
RPL39L: ribosomal protein L39 like
RPL39: ribosomal protein L39
CML: chronic myeloid leukemia
MX1: myxovirus resistance 1
GTPase: guanosine triphosphatas
HEXIM1: hexamethylene bisacetamide-inducible protein 1
BET: bromodomain and extra-terminal domain
MAP4K4: mitogen-activated protein kinase kinase kinase kinase 4
IL-18: interleukin-18
ICD: immunogenic cell death
LSC: leukemia stem cell
HSPC: hematopoietic stem and progenitor cell
HSC: hematopoietic stem cell
CpG: Cytosine-phosphate-Guanine
ns: non-significant
NES: normalized enrichment score

## Additional Information and Declarations

### Data Availability

The following information was supplied regarding data availability: The data is available at GitHub and Zenodo: - https://github.com/Yinghua-Dai/myeloid-cells-in-acute-myeloid-leukemia-raw-data
- Yinghua-Dai. (2026). Yinghua-Dai/myeloid-cells-in-acute-myeloid-leukemia-raw-data: Data-for-review (Version Raw-data) [Computer software]. Zenodo. https://doi.org/10.5281/zenodo.19870858
The raw sequence data are available at GenBank: GSE10212, GSE10358, GSE37642, GSE116256
